# Effectiveness of Nitazoxanide and Electrolyzed Oxiding Water in Treating Chagas Disease in a Canine Model

**DOI:** 10.3390/pharmaceutics15051479

**Published:** 2023-05-12

**Authors:** Olivia Rodríguez-Morales, Erika Jocelin Mendoza-Téllez, Elizabeth Morales-Salinas, Minerva Arce-Fonseca

**Affiliations:** 1 Laboratory of Molecular Immunology and Proteomics, Department of Molecular Biology of Instituto Nacional de Cardiología Ignacio Chávez, Juan Badiano No. 1, Col. Sección XVI, Tlalpan, Mexico City 14080, Mexico; rm.olivia@gmail.com (O.R.-M.); mendozatej24@gmail.com (E.J.M.-T.); 2 Department of Pathology of Facultad de Medicina Veterinaria y Zootecnia, Universidad Nacional Autónoma de México, Av. Universidad 3000, Col. Copilco Universidad, Coyoacán, Mexico City 04510, Mexico; moraless@unam.mx

**Keywords:** Chagas disease, *Trypanosoma cruzi*, electrolyzed oxidizing water, nitazoxanide, trypanocidal treatment, dilated cardiomyopathy, canine model

## Abstract

Chagas disease (CD) is caused by the protozoan *Trypanosoma cruzi*, and affects seven million people in Latin America. Side effects and the limited efficacy of current treatment have led to new drug research. The objective of this work was to evaluate the effectiveness of nitazoxanide (NTZ) and electrolyzed oxidizing water (EOW) in a canine model of experimental CD. Náhuatl dogs were infected with the *T. cruzi* H8 strain and NTZ- or EOW-treated orally for 10 days. Seronegativity was shown at 12 months post-infection (mpi) in the NTZ-, EOW-, and benznidazole (BNZ)-treated groups. The NTZ and BNZ groups had high levels of IFN-γ, TNF-α, IL-6, IL-12B, and IL-1β at 1.5 mpi and low levels of IL-10. Electrocardiographic studies showed alterations from 3 mpi and worsening at 12 mpi; NTZ treatment produced fewer cardiac pathomorphological changes compared to EOW, similar to BNZ treatment. There was no cardiomegaly in any group. In conclusion, although NTZ and EOW did not prevent changes in cardiac conductivity, they were able to avoid the severity of heart damage in the chronic phase of CD. NTZ induced a favorable proinflammatory immune response after infection, being a better option than EOW as a possible treatment for CD after BNZ.

## 1. Introduction

Chagas disease (CD), caused by the protozoan *Trypanosoma cruzi*, is one of the main causes of death due to heart failure—about 15 million people are currently estimated to be infected in Latin American endemic countries—and it has also become an important health issue in United States and Europe due to human migration [1,2]. In the acute phase of the disease in humans, clinical signs are usually mild, despite high levels of parasitaemia, which is characteristic of this phase of infection and which declines with the onset of immunity [3]. After this phase, individuals recover to an apparently healthy status, but a mild focal myocarditis may persist and may last for years, without signs of cumulative damage, in an asymptomatic chronic phase of the disease. Many years after the primary infection, about 30% of these individuals develop the symptomatic chronic phase, characterized by the presence of myocarditis and/or pathological disturbances in the peripheral nervous and gastrointestinal systems [4,5,6].

The current treatments, used for >40 years, nifurtimox and benznidazole (BNZ), have side effects—which force the patient to interrupt their therapeutic protocol—and limited cure efficacy (in acute phase, 50–80%; in chronic phase, 8–20%); additionally, the existence of *T. cruzi* strains naturally resistant to both drugs may partly explain the low cure rates detected in treated chagasic patients [7]. Therefore, there is a need to find novel drugs with trypanocidal activity that reduce the cardiac lesions developed in the chronic stage of CD. Nitazoxanide (NTZ) and electrolyzed oxidizing water (EOW) are under investigation as anti-trypanosome compounds, the first in the concept of drug repositioning and the second as a disinfectant with pharmacological use. NTZ is a broad spectrum antiparasitic belonging to the class of thiazolides, inhibit pyruvate ferredoxin oxidoreductase, which catalyzes the decarboxylation of pyruvate to acetyl coenzyme-A and CO_2_ with reduced equivalents transferred to ferredoxin or flavodoxin, enzymes of the energy metabolism of microorganisms. In diseases caused by helminths, extracellular anaerobic protozoa and bacteria, intracellular parasites and virus NTZ is indicated. NTZ antiparasitic activities in vitro have been reported on intestinal unicellular parasites (*Giardia intestinalis* and *Entamoeba histolytica*), the urogenital tract (*Trichomonas vaginalis*), hemoparasites (*Plasmodium berghei*) and kinetoplastid parasites such as *Trypanosoma cruzi* and *Leishmania mexicana* [8,9]. The NTZ therapeutic dose reported for mice against *T. cruzi* is 100 and 1000 mg/kg [10].

Likewise, EOW at neutral pH is currently used as a disinfectant to eliminate the growth of a variety of microorganisms, including bacteria, viruses, and fungi, without studies regarding effectiveness in protozoa. The way the EOW acts on microbes is attributed to oxidation of the sulfhydryl groups and amino acids of bacterial wall that affects the respiration and nutrition process, inhibition in the synthesis of proteins, breaking of the RNA chains and repression in the synthesis of cell metabolism molecules [11,12,13].

Since it is a disinfectant substance, there is no recommended therapeutic dose, nor has the mechanism of action through oral administration been fully elucidated; however, Lee et al. (2009) used an electrolyzed reduced water in a murine model of *Echinostoma hortense* infection and observed a local immunomodulatory effect by differential cytokine production [14]. In previous studies, the use of EOW as a treatment against experimental Chagas disease in mice showed its safety and effectiveness against *T. cruzi*, probably due to its indirect effect by modulating the immune response [15].

The establishment of experimental models that reflect the human disease and studies for specific treatment of CD are necessary. Experimentally infected dogs develop acute and chronic phases of chagasic infection that are comparable to clinical signs of the human disease [16]. Specifically, in the chronic stage, dogs exhibit a low intensity, progressive but incessant myocarditis, which allows alterations in the contractile function and heart dilation of the four chambers. Left ventricular apical aneurysm and other abnormalities of the wall of each cardiac segment are common. Histological examination shows disseminated destruction of cardiomyocytes, diffuse fibrosis, edema, mononuclear leukocyte infiltration in the myocardium and scarring of the heart conduction system. Progressive destruction of the cardiac fibers and intense fibrosis that replaces the cardiomyocytes lysis predisposes to cardiac failure and ventricular arrhythmia [17,18].

The present study aims to determine the effectiveness of NTZ and EOW in treating chronic chagasic heart disease in a canine model, by analysing their effect on cardiac pathologies.

## 2. Materials and Methods

### 2.1. In Vivo Model: Dog and Infection

Sixteen Náhuatl dogs [19] (the Náhuatl breed was verified, certified, and validated as a biological model by the Mexican Association of Laboratory Animal Science, A. C. since 2015) from 2 to 11 years old, both genders (12 males and 2 females), weighing between 14 and 27 kg, from the Animal Facility at the Instituto Nacional de Cardiología Ignacio Chávez (INCICh), were used. All dogs were confirmed negative to anti *T. cruzi* antibodies using standardized enzyme-linked immunosorbent assay (ELISA) before starting the study. The animals were divided randomly into six groups: non-infected and untreated control group (HEALTHY) (*n* = 2), *T. cruzi*-infected and NTZ-treated group (*Tc*/NTZ) (*n* = 4), *T. cruzi*-infected and EOW-treated group (*Tc*/EOW) (*n* = 4), *T. cruzi*-infected and BNZ-treated control group (*Tc*/BNZ) (*n* = 2), *T. cruzi*-infected and untreated group (*Tc*/UNT) (*n* = 2) and acute *T. cruzi*-infected and untreated group (*Tc*A/UNT) (*n* = 2). The dogs from five of the six groups were infected with a well-characterized Mexican H8 strain (MHOM/MX/1994/H8 Yucatán DTU I) of *T. cruzi* by intraperitoneal injection with 1.25 × 10^6^ blood trypomastigotes (resuspended in physiological saline) per animal that were obtained from infected bloodstream of BALB/c mice.

**Figure 1:**
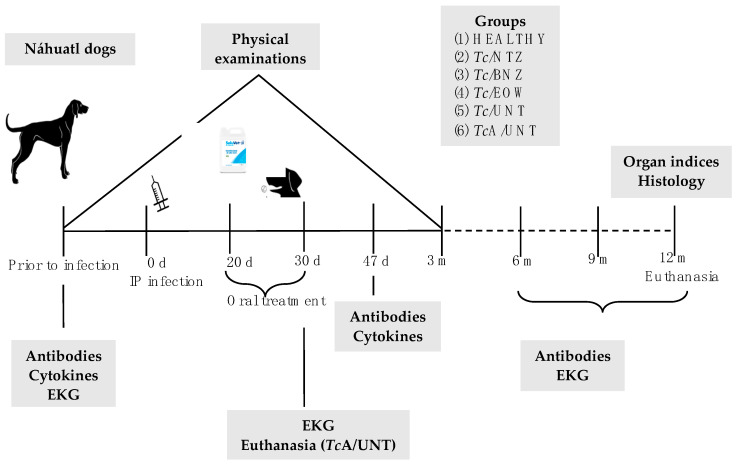
Schematic representation of the methodological design. Five groups of dogs were intraperitoneally infected with 1.25 × 10^6^ blood trypomastigotes of the H8 *T. cruzi* strain to evaluate the effectiveness of NTZ and EOW. IP = intraperitoneal, EKG = electrocardiogram, d = days, m = months.

All procedures carried out with dogs (Figure 1) were approved by the Bioethics Committee of the INCICh and the animal handling followed the established guidelines of the International Guiding Principles for Biomedical Research involving Animals and the Norma Oficial Mexicana (NOM-062-ZOO 1999) Technical Specifications for the Care and Use of Laboratory Animals [20].

### 2.2. Therapeutic Model

The dogs received the following drugs at therapeutic doses 20 days post-infection (dpi): NTZ (DAXON^®^, Siegfried Rhein, Querétaro, Mexico) at 50 mg/kg/day, EOW (SoluVet^®^, Esteripharma S.A. de C.V., Mexico City, Mexico) 200 mL at 60 ppm/animal/day, and BNZ at 40 mg/kg/day (LAFEPE^®^, Laboratório Farmacéutico do Estado de Pernambuco S/A, Brazil); all of them were orally administered for ten consecutive days. No treatment was given to heathy control group. Only Daxon^®^ was purchased commercially from a drug store; while Soluvet^®^ and Lafepe^®^ were given as a donation.

### 2.3. Antibodies Detection

For detection of anti-*T. cruzi* immunoglobulin G (IgG) pre-infection and at 1.5, 3.5, 9.5 and 12-months post-infection (mpi), the dog serum samples were processed in duplicate and analyzed by the ELISA method using a whole protein extract of *T. cruzi* INC-9 isolate as antigen as described previously [21,22]. Briefly, 96 Maxisorb plates (Nunc^®^, ThermoFisher Scientific, Waltham, MA, USA) and peroxidase conjugated anti-dog IgG antibody (Novus Biologicals^®^, Bio-Techne Corporation, Minneapolis, MN, USA) were employed. Absorbance values were determined at 495 nm in a Microplate Reader (Bio-Rad^®^, Bio-Rad Laboratories, Inc., Hercules, CA, USA). The cut-off value was obtained from a negative control serum plus three standard deviations (S.D.). The data presented are the mean of the values for each dog.

### 2.4. Cytokines Determination

Interleukine-1β (IL-β), IL-6, IL-10, IL-12B, Interferon-γ (IFN–γ) and Tumoral Necrosis Factor-α (TNF-α) levels in sera from all dogs prior to infection, 47 dpi and 15 days post-treatment (dpt) were measured by ELISA according to the supplier’s specifications (Biotechne Brand^®^, Bio-Techne Corporation, Minneapolis, MN, USA; Aviva Systems Biology^®^, Aviva Systems Biology, Corp., San Diego, CA, USA and Invitrogen^®^, ThermoFisher Scientific, Waltham, MA, USA commercial kits).

### 2.5. Electrocardiography

Electrocardiographic evaluations included those performed before infection and 3, 6, 9, and 12 mpi. The tracings were recorded by three standard bipolar leads (I, II, and III); three unipolar augmented leads (AVR, AVL, and AVF) and four unipolar precordial leads (CV5RL, CV6LL, CV6LU, and V10); the voltage was standardized to 1 mV/cm at a 50 mm/s paper speed using a Schiller AT-1^®^ (Shiller Americas México S. A. de C. V., Mexico City, Mexico) machine. No sedative for movement restriction during the procedure was used. The electrocardiograms (EKG) of all dogs were recorded in the right lateral decubitus position and on a non-metallic surface, as described by Tilley (1979) [23].

### 2.6. Euthanasia and Heart Pathological Findings

Euthanasia method was carried out at 12 mpi in all groups, with the exception of the *Tc*A/UNT group, which was at 30 dpi, according to the Norma Oficial Mexicana NOM-033-SAG/ZOO-2014 Methods to Bring Death upon Domestic and Wild Animals [24] by intravenous injection of pentobarbital sodium (Dolethal^®^, Vetoquinol de México, S. A. de C. V., Mexico City, Mexico) at a lethal dose of 90 at 210 mg/kg and potassium chloride at a concentration of 149 mg/mL/total dose. The macroscopic description of the alterations in the heart was performed based on the methods described by de Aluja (2002) [25].

### 2.7. Evaluation of the Cardiac, Splenic and Lymphonodal Indices

Prior to euthanasia, body weight was recorded using an Omron^®^ (Omron México, Mexico City, Mexico) digital scale and the weight of the heart, the spleen, and the popliteal lymph nodes were obtained using a Sartorius^®^ (Sartorius de México S. A. de C. V., Tepotzotlán, Mexico) BL1500S digital scale during the necropsy procedure. Cardiomegaly, splenomegaly and lymphadenopathy were determined with their respective indices (organ weight/body weight × 100). Cardiomegaly, splenomegaly, and lymphadenopathy were considered when the organ index was significantly higher (*p* ≤ 0.05) than that observed in the organs from negative control, healthy, non-infected dogs [26].

### 2.8. Heart Histology

Longitudinal and transversal right ventricle (RV) and left ventricle (LV) heart muscle tissues were obtained. Tissue sections were fixed in 10% buffered formalin for 24 h. Samples were dehydrated in absolute ethanol, cleared in xylene and embedded in paraffin. Non-contiguous sections at 5 μm thickness were cut and stained with haematoxylin and eosin. Cellular infiltrates were examined by light microscopy (Leica^®^, Leica CME, Wetzlar, Germany) and images were captured with a Spot RT^®^ (Diagnostic Instruments, NY, USA) digital camera. At least 20 random microscopic fields (100 and 400×) were analyzed in each microscopic section using the open source Image J software (NIH, Beteshda, MD, USA).

### 2.9. Statistical Analysis

Data were assessed for normal distribution by Kolmogorov–Smirnov (K–S) test and checked by histograms and Q-Q plots. The results were expressed as the means ± S.D. Antibodies determination, cytokines levels, and organ indices were analyzed by the Kruskal–Wallis test (SPSS software, version 13.0). The H *post-hoc* test was performed to comparison of multiple groups. Differences were considered statistically significant when *p* < 0.05.

## 3. Results

### 3.1. Evidence of Establishment of Infection

Presence of parasites in blood not was detected by light microscopy (parasitaemia) in all infected groups by examining freshly isolated blood samples collected between 22 and 55 dpi; however, the establishment of infection was confirmed in all experimental infected groups: *Tc*/NTZ, *Tc*/EOW, *Tc*/BNZ, *Tc*/UNT, and *Tc*A/UNT by specific IgG anti *T. cruzi* antibodies detection by ELISA and IIF serological tests at 47 dpi.

### 3.2. Detection of IgG Antibodies

**Figure 2:**
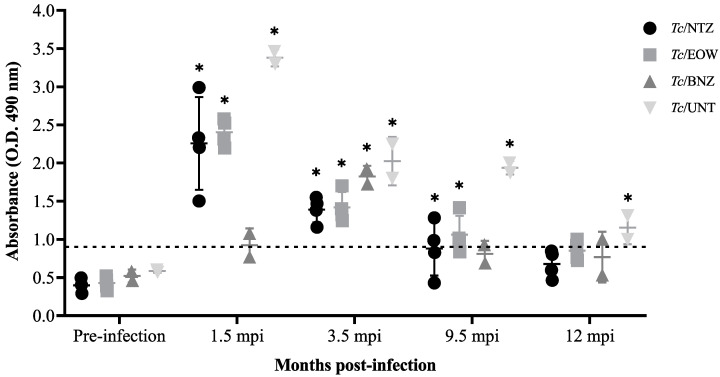
Immunoglobulin G levels in sera of Náhuatl dogs experimentally infected with *T. cruzi* and untreated or treated with NTZ, EOW, or BNZ. The values represent the means of duplicate assays ± S.D. for detection of IgG. The dotted line shows the cut-off value in the ELISA. Each group was compared with the cut-off value and differences were considered significant (*) at *p* ≤ 0.05 by using Kruskal–Wallis statistical test.

At 1.5 mpi (15 dpt), 3.5 mpi (2.5 mpt), and 9.5 mpi (8.5 mpt), the *Tc*/NTZ, *Tc*/EOW, and *Tc*/UNT groups were immunopositive to parasite infection by the presence of IgG anti-*T. cruzi* antibodies. From 9.5 mpi, the control group treated with BNZ was seronegative, and so it remained during the subsequent determinations due to the already-proven trypanocidal action of this drug. At 12 mpi/11 mpt, the *Tc*/NTZ and *Tc*/EOW groups showed negative seroconversion, while the individuals of the *Tc*/UNT group persisted as positive. Although there was variation in the different groups in the level of antibody production, the results shown indicate that the experimental treatments with NTZ or EOW seem to have a curative effect by eliminating the parasites and, therefore, there was no longer an immunogenic response (generated by the presence of the parasite), as does the BNZ treatment (Figure 2).

### 3.3. Determination of Cytokines

**Figure 3:**
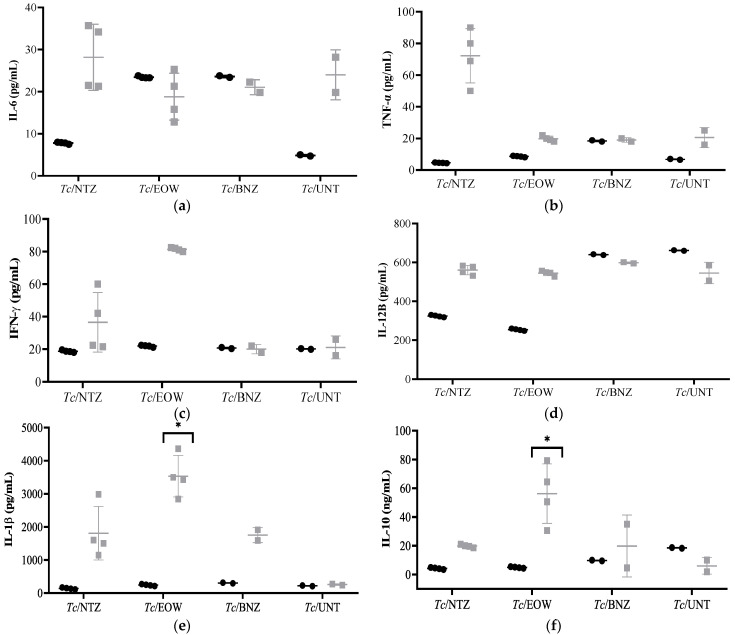
Serum cytokines levels in a canine model of infection with *T. cruzi* to evaluate the effect of NTZ and EOW. (**a**) IL-6, (**b**) TNF-α, (**c**) IFN-γ, (**d**) IL-12B, and (**e**) IL-1β were detected at pg/mL, while (**f**) IL-10 was in units of ng/mL. Treatment was administered for 10 days starting at 20 dpi. Each bar shows the average concentration (±S.D.) of the different cytokines. Each group was compared with the *Tc*/UNT group and differences were considered significant (*) at *p* ≤ 0.05 by using Kruskal–Wallis statistical test. dpi = days post-infection; dpt, days post-treatment. Black circles = pre-infection, and grey squares = 47 dpi/15 dpt.

Although the statistical analysis did not reflect significant differences in the levels of most of the cytokines evaluated, different behaviors were observed among groups. The *Tc*/NTZ group showed a clear increase in the response of pro-inflammatory cytokines: IL-6 (Figure 3a), TNF-α (Figure 3b), IFN-γ (Figure 3c), IL-12B (Figure 3d), and IL-1β (Figure 3e), with a moderate response of IL-10 (Figure 3f). The *Tc*/EOW group produced a response with a predominance of IFN-γ and IL-12B, and significantly high IL-1β (*p* = 0.0152) and IL-10 (*p* = 0.0247), showing the highest concentration at the time of infection of this last cytokine. The *Tc*/BNZ group did not have a predominance of IFN-γ, TNF-α, IL-6, or IL-12B, but was characterized by an IL-1β increase. Finally, the *Tc*/UNT group induced a Th1 response mainly with high levels of IL-6, followed by IFN-γ and TNFα. As expected, the non-infected and untreated group (HEALTHY) had concentrations close to baseline values (not plotted). These results show that the experimental treatments with NTZ and EOW had a different effect on cytokine kinetics than that of the control groups with BNZ or without treatment, so that this immune response could change the course of the disease and its resolution.

### 3.4. Electrocardiographic Alterations

**Figure 4:**
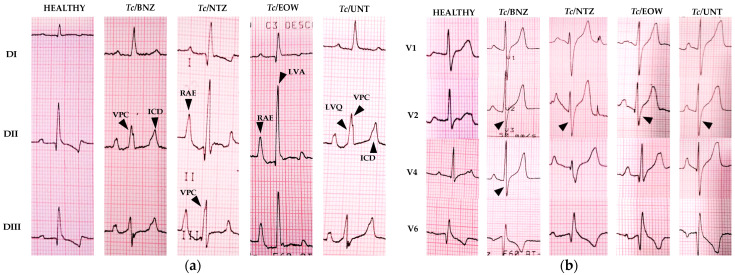
Electrocardiographic findings in a canine model of *T. cruzi* infection for evaluation of treatment with NTZ or EOW at 12 mpi (11 mpt). (**a**) Standard limb leads, in which arrowheads show: HEALTHY, without alterations; *Tc*/BNZ, ventricular premature complex (VPC), and intraventricular conductivity defect (ICD); *Tc*/NTZ, right atrial enlargement (RAE), and ventricular premature complex (VPC); *Tc*/EOW, right atrial enlargement (RAE), and left ventricular enlargement (LVA); *Tc*/UNT, low voltage QRS complex (LVQ), ventricular premature complex (VPC), and intraventricular conduction defect (ICD). (**b**) Precordial leads, in which arrowheads point to changes suggestive of right bundle branch block (RBBB); the S wave is wider in the QRS complex in V2 and/or V4 leads, together with other changes in different leads. Paper speed 50 mm/s, voltage 1 mV/cm.

Although the statistical analysis did not reflect significant differences in the EKG tracings, a different behavior of them was observed among groups. Electrocardiographic findings of the canine model of infection with *T. cruzi* for the evaluation of treatment with NTZ and EOW are shown in Figure 4 and Table 1.

The *Tc* /NTZ group showed alterations in conductivity, mainly at 3 mpi (2 mpt); these alterations consisted of ventricular premature complexes (morphological alteration of the R wave), defect in intraventricular conduction (alteration in the T wave), and low QRS voltage (amplitude in QRS less than 0.5 mV), as well as enlargement of the right atrium (increase in the amplitude and morphological abnormality of the P wave) and alterations suggestive of right bundle branch block (very deep S wave) in one individual at 12 mpi (11 mpt).

The *Tc*/EOW group exhibited persistent alterations from 3 mpi (2 mpt) and an increase in the severity and intensity of the lesions at 12 mpi (11 mpt) in 50% to 75% of the individuals in this group. Findings included right atrial enlargement (increase in the amplitude and morphological abnormality of the P wave), left ventricular enlargement (increased duration of the QRS complex and high-amplitude R wave), low voltage in the QRS complex (QRS amplitude less than 0.5 mV), intraventricular conduction defect (alteration in the T wave) possibly associated with right or left bundle branch block, ventricular premature complex (alteration in R wave morphology), and right bundle branch block (very prominent S wave).

The EKG alterations related to the failure in conductivity in the *Tc*/BNZ group are similar to those described for the *Tc*/EOW group; however, the dogs of that group had no enlargement of the cardiac chambers recorded by the EKG.

The individuals in the *Tc*/UNT group exhibited only morphological or polarity alterations of the T wave: intraventricular conduction defect (with the possibility of association with right or left bundle branch block) and errant sinus pacemaker. The negative control group (HEALTHY) did not register pathological changes in the EKG.

### 3.5. Heart Anatomopathological Alterations to Necropsy

**Figure 5:**
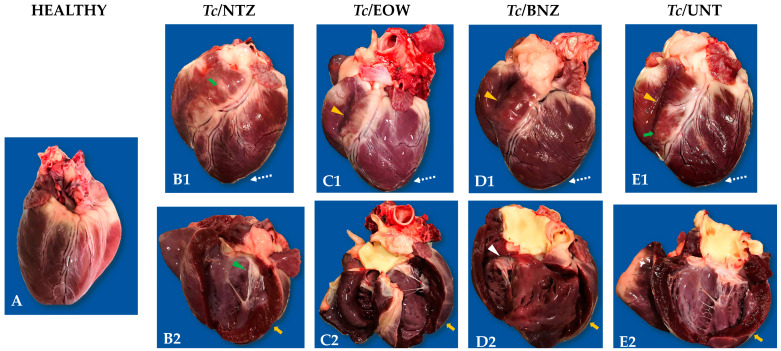
Macroscopic cardiac characteristics obtained from the canine model of infection with *T. cruzi* to evaluate the effect of NTZ and EOW. (**A**). Heart of a clinically healthy animal, without alterations. (**B1**,**B2**). Infected and NTZ-treated dog heart, silhouette and longitudinal section in left ventricle, respectively. Tabby pattern (green arrow) in right ventricle, rounded silhouette (dotted arrow), mitral endocardiosis (green arrowhead), and left ventricular hypertrophy (orange arrow). (**C1**,**C2**). Infected and EOW-treated dog heart, silhouette and longitudinal section in right ventricle and left ventricle, respectively. Rounded silhouette (dotted arrow), right ventricular dilatation (orange arrowhead) with left ventricular hypertrophy (orange arrow). (**D1**,**D2**). Infected and BNZ-treated dog heart, silhouette, and longitudinal section in right ventricle, respectively. Rounded silhouette (dotted arrow), right ventricular dilatation (orange arrowhead), and tricuspid endocardiosis (white arrowhead). (**E1**,**E2**). Infected and untreated dog heart, silhouette and longitudinal section in left ventricle, respectively. Rounded silhouette (dotted arrow), tabby pattern (green arrow), right ventricular dilatation (orange arrowhead) with left ventricular hypertrophy (orange arrow).

A rounded cardiac silhouette, paleness epicardium, dilatation of the RV and LV, tricuspid and mitral valves endocardiosis, a tabby pattern in epicardium, and fluid pericardium accumulation at the macroscopic examination (12 mpi) in the *Tc*/UNT group were the main lesions found (Table 2 and Figure 5). The *Tc*/NTZ and *Tc*/EOW groups (11 months post treatment) exhibited all of the findings described, in 25% to 75% and 25% to 100%, respectively (Table 2). There were no notable differences compared to the *Tc*/BNZ group, except for the development of bivalvular endocardiosis. The healthy control group did not show morphopathological cardiac abnormalities.

However, the severity of the lesions in the *Tc*/UNT is apparent greater than in any experimental group infected with treatment, in that sense that treatment with NTZ or EOW is functional for the progress or severity of chagasic cardiomyopathy.

### 3.6. Heart, Splenic and Popliteal Lymph Node Indices

**Figure 6:**
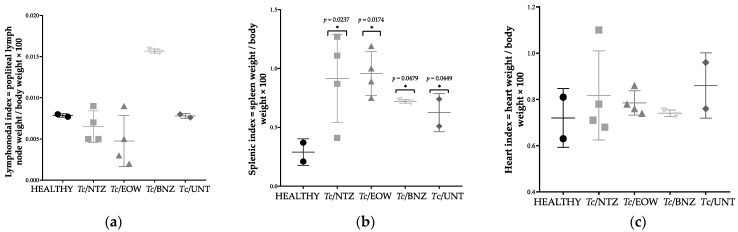
Organ indices during chronic stage of *T. cruzi* infection in Náhuatl dogs treated with NTZ or EOW. (**a**) Lymhpadenopathy, (**b**) splenomegaly and (**c**) cardiomegaly. Each group was compared with the HEALTHY group and differences were considered significant (*) at *p* ≤ 0.05 by using Kruskal-Wallis statistical test.

In order to determine whether cardiomegaly, splenomegaly, and lymphadenopathy had occurred in infected and infected/treated dogs, the organic indices were calculated. The popliteal lymph node index was evaluated and no statistically significant difference (*p* = 0.292) was obtained to consider lymphadenopathy in the groups treated with NTZ or EOW (Figure 6a). However, it is possible to observe a high lymph node index in the dogs treated with BNZ, which suggested probable reactive hyperplasia.

According to the mains obtained for the splenic indices, a statistically significant difference (*p* = 0.034) was demonstrated to consider splenomegaly—which is a common sign of CD—in the *Tc*/NTZ, *Tc*/EOW, *Tc*/BNZ, and *Tc*/UNT groups with respect to the HEALTHY group (Figure 6b).

Although the heart index means of the *Tc*/UNT, *Tc*/NTZ, and *Tc*/EOW groups did not show significantly values above normal, one dog from each group developed cardiomegaly, representing 50% for *Tc*/UNT and 25% for *Tc*/NTZ and *Tc*/EOW (Figure 6c). This result indicated that the experimental treatments with NTZ or EOW were able to prevent cardiomegaly to the same extent as benznidazole—the drug of choice in the treatment of CD. 

### 3.7. Heart Histological Findings

**Figure 7:**
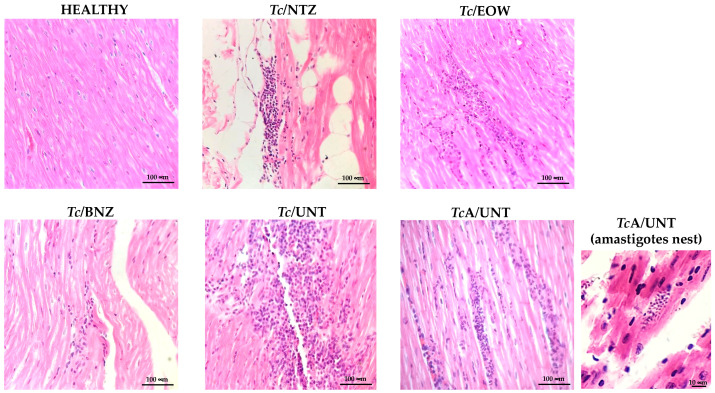
Histological findings in a longitudinal section of the left ventricle of the heart in the canine model of infection with *T. cruzi* for the evaluation of the effect of NTZ and EOW. Representative micrographs of each group: HEALTHY, without histological alterations; *Tc*/NTZ, focal mild interstitial lymphoplasmacytic myocarditis in epicardium; *Tc*/EOW, moderate multifocal coalescent lymphocytic, plasmocytic, and eosinophilic myocarditis; *Tc*/BNZ, mild multifocal interstitial lymphoplasmacytic myocarditis and elongation of muscle fibers; *Tc*/UNT, multifocal moderate to severe coalescent lymphoplasmacytic myocarditis; *TcA*/UNT, multifocal moderate segmental lymphoplasmacytic myocarditis and presence of amastigotes nests in another histological section. Hematoxylin and eosin staining, visualized by light microscopy (400× magnification). The evaluation was carried out at 12 mpi (11 mpt), with the exception of the *TcA*/UNT group, which was studied at 30 dpi.

The HEALTHY group did not show histological myocardial abnormalities. Lymphoplasmacytic and eosinophilic lymphoplasmacytic interstitial ventricular myocarditis was observed in all infected groups (Figure 7); the degree of severity is variable depends on the experimental group. The *Tc*/NTZ, *Tc*/EOW, and *Tc*/BNZ groups showed mild inflammation; an individual from the *Tc*/BNZ group showed moderate myofibrillar degeneration. An individual from the *Tc*/EOW group exhibited moderate to severe segmental inflammation. The *Tc*/UNT group presented severe coalescent inflammation. There was no evidence of fibrosis, edema, or intracellular amastigotes nests in any of these groups.

The *TcA*/UNT group had moderate multifocal inflammation with some nests of intracellular amastigotes in the myocardium, a finding that is widely recognized in the acute stage of experimental CD.

These findings suggest that tested treatments controlled the infection in different magnitudes and consequently reduced the inflammatory infiltrate responsible for cardiomyopathy; treatment with NTZ presented results similar to those of BZN.

## 4. Discussion

In the last 10 years, only one research paper has been published that addresses the use of NTZ as a treatment for CD, in which Valle-Reyes et al. [10] infected BALB/c mice with the Mexican *T. cruzi* Albarrada strain and orally administered nifurtimox or NTZ (100 mg/kg and 1000 mg/kg; Daxon^®^) or both drugs for 16 days; they found that parasitaemia, tissue damage, and mortality caused by *T. cruzi* infection were significantly higher in the NTZ-treated groups of mice, even compared to untreated infected animals. The authors concluded that NTZ cannot be used as a treatment for CD [10]. However, our results in the canine model are the opposite. It is probable that the use of the Mexican H8 strain and the treatment scheme, in which NTZ was used for 10 days at a dose of 50 mg/kg, were decisive.

It is important to highlight that NTZ is not for veterinary use; therefore, there is no established dose for this drug in this animal species. On the other hand, the doses in mice used by Valle-Reyes et al. [10] far exceeded that used in humans, which is 7.5 mg/kg BID (twice a day). It was decided to use a dose of 50 mg/kg SID (once a day) to avoid toxicity while still being higher than that used against intestinal parasites.

On the other hand, the use of electrolyzed water has been widely extended in various fields such as wound healing, advanced tissue care, dental clinics [27], disinfection of hospital areas [28], food sterilization [29], livestock industry [30], and pharmacological use [31]. New applications have also been found for electrolyzed water, such as the treatment of municipal solid waste [32]. The uses of the EOW have been limited to the elimination of fungi, bacteria, and viruses; however, its use as a parasiticide has not been explored, since there are no published reports in this regard, except for those of our group, in which we focused on its use as a trypanocide in animal models of CD and hymenolepiosis (manuscripts in preparation).

In an effort to improve the effectiveness of BNZ, it has been tested in combination with other drugs [33,34] or it has been administered in different presentations, such as micro- and nanoparticles [35,36]; in addition, other molecules have been used as alternative treatments [37,38,39]. The prevailing need to find a targeted treatment for CD that is effective in the acute and chronic stages, mainly against the cardiac presentation, and that does not have serious side effects, led to the study of NTZ and EOW as treatment in the canine model.

The cardiomyopathy caused by parasite-specific immune responses to *T. cruzi* parasites or antigen persistent in the heart, parasite mediated myocytolysis, primary neuronal damage, damage to cardiac microvasculature, is the most common clinical presentation in the chronic phase of CD [40], whose current treatment has low effectiveness [41]. NTZ is a low-cost drug with antiprotozoal, antibacterial, and antiviral activity that has been reported against *T. cruzi* epimastigotes in vitro [8]. Similarly, EOW is a disinfectant directed to bacteria, fungi, and viruses, but has not yet been tested in parasites [13], which suggests that it may have trypanocidal activity as well. Therefore, in the present study, the canine model was used due to the similarity of CD pathogenesis development compared to humans [21,42,43], to test the effectiveness of NTZ and EOW in treating chronic chagasic heart disease by analysing their effect on cardiac pathologies.

Dog mortality was not observed in either group throughout the research work, unlike in other studies, in which experimental *T. cruzi* inoculation caused sudden death in some of the dogs. However, in those studies, mongrel dogs were used, with a different number of animal samples, with another *T. cruzi* strain or morphologic form of the parasite, and with different inoculum size or different administration routes [44,45,46]. Our experimental CD canine model has been standardized in accordance with median lethal dose (LD_50_ test) assays to determine an adequate inoculum and to achieve the establishment of the acute and chronic phases of CD through the systemic infection.

Cunha et al. [34] evaluated dogs infected with a BNZ-resistant *T. cruzi* strain and treated with BNZ or itraconazole (ITZ) or with both drugs for 60 days. Polymerase chain reaction (PCR) was negative in 2 of 3 animals in the BNZ + ITZ group, 2 of 5 in the BNZ group, and 4 of 5 in the ITZ group; the blood culture performed at month 24 was negative in all groups and the serum samples evaluated by ELISA remained reactive in all treated animals [34]. In contrast, in the present study, the detection of IgG antibodies indicated a specific anti-*T. cruzi* immune response from 3 mpi to 9.5 mpi. At 12 mpi, the negative seroconversion can be interpreted as an absolute decrease in the antibody production stimulus with exclusive storage of memory antibodies [13,47]. The goal of successful treatment is to gradually decrease antibody titers in serological diagnostic tests [48]. The administration of BNZ, NTZ, and EOW in this experimental model induced seronegativization, which indicates the parasite elimination from the organism and, therefore, a parasitological cure at 11 mpt (12 mpi), a parameter that provided effectiveness in favor of the experimental treatments. The discrepancy in the results with these other authors may be largely due to the strain of *T. cruzi* used in each study.

The *Tc*/UNT group showed a response mediated mainly by IL-6, IFN-γ, and TNF-α, with no IL-10 response during acute infection at 47 dpi; the group with the reference treatment (BNZ) responded at 15 dpt in a very similar way to the group without treatment, which is consistent with what was reported by other authors [49]. The IL-1β production in the groups that received some treatment is highlighted, as reported by Piaggio et al., who point out that IL-1β exhibits proinflammatory activity during BNZ treatment, as well as it is involved in immune protection by intracellular pathogens infections; therefore, IL-1β is indicative of an effective adaptive immune response [50].

The cellular immune response presented by the *T*c/NTZ group at 15 dpt and 47 dpi is capable of deterring *T. cruzi* infection, since it responded with high levels of TNF-α, followed by IL-6, IL-12B, IL-1β, and IFN-γ, and a discrete control of IL-10; therefore, compared to the previously discussed groups (*T*c/UNT and *Tc*/BNZ), the Th1 proinflammatory response was more intense. This cytokine profile should have triggered phagocytic cell activation and inflammation; TNF-α is synergistic with IFN-γ and amplifies nitric oxide production to contribute to parasite growth control. The fact that there was an IL-10 response indicated that there was a regulatory role in the intense inflammatory and immune response of the Th1 response, in agreement with other authors [51,52,53].

The predominance of the proinflammatory Th1 profile creates an immunological paradox, since despite the trypanocidal immune reaction, it allows cardiac presentation and residual parasitism, which are present in the chronic phase of CD [54]. In the *Tc*/BNZ group, the degree of severity of myocarditis and its sequelae decreased, and in the group treated with NTZ, it is suggested that it had the same or better effect.

In the *Tc*/EOW group, an unpolarized immune response was demonstrated, since a Th1/Th2 balance was maintained. It exerted a strong response with IFN-γ, IL-1β, and IL-12B, resulting in a decrease in parasitaemia and tissue parasitosis, as has been reported by other authors [55,56]; however, the elevated concentration of IL-10 could have several explanations: preventing immune hyperactivity during infection [57], limiting the parasite load, and protecting against fatal myocarditis [58], or improving susceptibility to infection with *T. cruzi* with a fatal cardiac presentation [59].

Electrocardiographic changes in the *Tc*/NTZ and *Tc*/EOW groups–from three to 11 mpt–included right atrial enlargement associated with conduction disturbances from the sinoatrial node to the atrioventricular node, left ventricular enlargement by probable concentric hypertrophy due to volume overload, low QRS voltage associated with myocardial damage or pericardial fluid accumulation, intraventricular conduction defect due to incomplete right bundle branch block or ventricular enlargement, ventricular premature complex related to congestive heart failure, and complete right bundle branch block due to thickening of the ventricular wall. In the *Tc*/BNZ and *Tc/*UNT groups, the abnormalities were poorly differentiated bundle branch block with intraventricular conduction defect. All findings are consistent with what has been reported in dogs with CD [4,6,42,60,61,62]. There is a possibility that NTZ may have caused cardiotoxicity, as reported by Gong et al., who observed that exposure to the drug affected the embryonic heart development of zebrafish, significantly inhibited cardiomyocyte proliferation, and promoted apoptosis, ultimately leading to cardiac tissue injury [63]. Regarding EOW, this compound has not been reported to aggravate pathological conductive presentations in the heart as part of its acute and chronic side effects [64].

Heart index to determine cardiomegaly did not show statistically significant differences in groups treated with NTZ, EOW, or BNZ compared to the HEALTHY group; therefore, cardiomegaly was not determined, despite the fact that left ventricular wall thickening was observed in all infected experimental groups. Only the group of dogs that received no treatment showed cardiomegaly by a high heart index; this is consistent with the results of other authors who found that in canine chronic CD, cardiomegaly occurs as part of the cardiac presentation due to myocardial tissue tropism, and consequently, different degrees of myocarditis and left ventricular thickening can be observed [16]. The disease progression time in the canine model of the present study was up to 12 mpi, which is sufficient time for the development of chronic CD, since it has been reported that clinical chronic CD in dogs can present eight months after infection [65].

Histopathological findings showed that NTZ and BNZ treatment induced mild focal mononuclear myocarditis, unlike what was reported for NTZ in the murine model of infection by Valle-Reyes et al., who reported moderate to severe myocarditis with myocyte destruction and mononuclear and fibroblastic infiltration in cardiac tissue [10]. As expected, the *T*c/UNT group exhibited severe multifocal and coalescing inflammation. Tissue inflammation is caused by *T. cruzi* infection due to the influence of proinflammatory cytokine activity such as IFN-γ, IL-12, and TNF-α and low activity of regulatory cytokines such as IL-4 and IL-10 [54], a situation that correlates with the cytokine profiles obtained in the present study. On the other hand, although the treatment with EOW also failed to reduce the degree and distribution of inflammation as did BNZ, the severity of the lesions was less than that of the group that received no treatment.

No amastigotes nests were observed in any section of the heart in any experimental group in the chronic stage. This is consistent with studies in dogs, mice, and humans, in which, amastigotes were only found in the acute phase of the disease [4,60,66,67,68,69,70], as observed in the *TcA*/UNT group. However, tissue parasitism can be detected with parasite DNA identification tests in inflammatory lesions using molecular tests such as PCR; or by immunohistochemistry, as reported by Higuchi et al., who found evidence of *T. cruzi* in 69% of 16 patients with chronic CD and the antigen was detected in 70% of heart sections with moderate or severe myocarditis but less than 17% in heart sections with mild or no myocarditis [71].

Areas of opportunity to complement the present study are the following: to test the effectiveness of the pharmacological mixtures of NTZ and BNZ, EOW and BNZ, as well as NTZ and EOW to evaluate possible synergistic effects. In regard to the humoral and cellular immune response mechanism research, the study can be enriched by evaluating the Th17 profile and other cytokines such as IL-4 and transforming growth factor-beta (TGF-β) to address the participation of other molecules that are involved in the susceptibility or protection to the disease. Work can also be performed to determine clinical parameters and cardiac biomarkers that can help assess the effects of NTZ and EOW in the treatment against canine CD.

## 5. Conclusions

The experimental NTZ and EOW treatments showed therapeutic efficacy similar to that of the drug of choice (BNZ) against the experimental canine infection by *T. cruzi*. Adequate pro-inflammatory immune response was better post-infection with NTZ treatment than with EOW. Experimental treatments did not prevent cardiac conductivity abnormalities, but could help to decrease the magnitude of cardiac lesions and severity of heart inflammation in the chronic stage of CD. This research is the first to describe the application of both NTZ or EOW compounds orally in dogs as a treatment against CD, with different degrees of effectiveness on chronic chagasic cardiomyopathy, showing similar results to BNZ, but without its toxic effects. These compounds—NTZ or EOW—could be considered as good therapeutic options in pets and working dogs, since there are no drugs against this disease for veterinary use. This contributes to expanding the range of possibilities for the use of alternative medicines to fight CD.

## Figures and Tables

**Table 1:** Electrocardiographic findings of the canine model of infection with *T. cruzi* for the evaluation of treatment with NTZ or EOW.

Experimental Group	mpi	Number of Individuals/n (%)	Electrocardiographic Interpretation
*Tc*/NTZ	3	3/4 (75)	WAP, ICD, VPC, HMI, LVQ
	6	2/4 (50)	WAP, HMI
	9	2/4 (50)	ICD
	12	1/4 (25)	RAE, ICD, VPC
*Tc*/EOW	3	2/4 (50)	ICD, HMI
	6	2/4 (50)	ICD, HMI
	9	2/4 (50)	AFDC, ICD
	12	3/4 (75)	RAE, LVE, ICD, RBBB
*Tc*/BNZ	3	1/2 (50)	ICD, LVQ, RBBB
	6	1/2 (50)	ICD
	9	2/2 (100)	ICD, HMI
	12	1/2 (50)	VPC, ICD, RBBB
*Tc/*UNT	3	2/2 (100)	ICD
	6	2/2 (100)	WAP, ICD
	9	2/2 (100)	WAP
	12	2/2 (100)	WAP, ICD

mpi = months post-infection; n = sample size. AFDC = atrial fibrillation due to dilated cardiomyopathy; HMI = hypoxia/myocardial infarction; ICD = intraventricular conduction defect; LVE = left ventricular enlargement; LVQ = low voltage in QRS; RAE = right atrial enlargement; RBBB = right bundle branch block; VPC = premature ventricular complex; WAP = wandering atrial pacemaker. The HEALTHY group is not included because it did not present alterations in the EKG during the development time of the study. The experimental groups did not present alterations in the EKG at the time of pre-infection.

**Table 2:** Percentage and proportions of the morphopathological alterations in the heart present in dogs infected with *T. cruzi* and treated with NTZ or EOW.

Morphological Alterations	*Tc*A/UNT % (No. of Individuals/*n*)	*Tc*A/UNT % (No. of Individuals/*n*)	*Tc*A/UNT % (No. of Individuals/*n*)	*Tc*A/UNT % (No. of Individuals/*n*)	*Tc*A/UNT % (No. of Individuals/*n*)
Rounded heart silhouette	0 (0/2)	50 (2/4)	75 (3/4)	100 (1/1)	100 (1/1)
Epicardial paleness	0 (0/2)	25 (1/4)	75 (3/4)	100 (1/1)	0 (0/1)
Right ventricular dilation	50 (1/2)	25 (1/4)	50 (2/4)	100 (1/1)	100 (1/1)
Left ventricular hypertrophy	100 (2/2)	75 (3/4)	75 (3/4)	100 (1/1)	100 (1/1)
Endocardiosis of the tricuspid valve	0 (0/2)	0 (0/4)	100 (4/4)	0 (0/1)	100 (1/1)
Endocardiosis of the mitral valve	50 (1/2)	25 (1/4)	75 (3/4)	0 (0/1)	100 (1/1)
Tabby pattern	0 (0/2)	25 (1/4)	25 (1/4)	0 (0/1)	100 (1/1)
Serosanguineous pericardial fluid	100 (2/2)	50 (2/4)	25 (1/4)	100 (1/1)	0 (0/1)

The evaluation was carried out at 12 mpi (11 mpt), with the exception of the *Tc*A/UNT group, which was studied at 30 dpi.

## Data Availability

All data is contained within the article. For any additional information, please contact the corresponding author.
